# A systems approach to developing a port community system for South Africa

**DOI:** 10.1186/s41072-022-00128-3

**Published:** 2022-11-30

**Authors:** Sphiwe Eugene Mthembu, Mihalis Georgiou Chasomeris

**Affiliations:** 1 Port of Durban Marine Services, Transnet National Ports Authority, Quayside Road, Durban, 4000 South Africa; 2 Graduate School of Business and Leadership, University of KwaZulu-Natal, Durban, South Africa

**Keywords:** Port community systems, Soft systems methodology, Maritime, Ports, Information systems, Supply chain, Integration

## Abstract

Port community systems (PCS), as electronic platforms enabling the intelligent and secure exchange of information between public and private stakeholders in ports, are central to port supply chains. PCS drive productivity, efficiencies, and competitiveness whilst improving the port’s attractiveness. They connect port users and supply chain participants and allow them to share information efficiently on a single platform by drawing data from different enterprise resource management systems. Port systems are complex networks of functions integrated to deliver cargo services to port users. Developing countries’ ports have suffered adversely from their slow adoption of PCS; consequently, their performance improvements have stagnated over time. This article uses Soft Systems Methodology and applies it to the case of South Africa’s ports that are particularly affected by the absence of PCS. The article also presents a framework for implementing PCS. Primary data was gathered through 24 interviews and two port stakeholder workshops. The findings show that port users are operating on fragmented and different platforms, lack integration and collaboration, and no single platform is used to share critical shipping information. Nevertheless, the interviewees all supported the creation of a PCS, and this article develops and recommends a framework for implementing a PCS in South Africa.

## Introduction

Significant challenges about procedural complexities, redundancy, unintegrated information, and document flow beset ports worldwide and impact various port users involved in seaport operations. These factors affect the port’s performance resulting from emergent complexities manifesting in delays in shipping (Varbanova, 2017). Emergent information technologies present ports with the opportunity to improve their competitiveness. Adopting emergent technologies and improved automation has severely reduced bottlenecks in the port supply chain systems (UNCTAD, 2017).

A substantial number of ports in developed countries have adopted PCS to take advantage of the system’s ability to integrate information about merchant shipping cargo. However, the situation differs in developing countries. According to Keceli (2011), most ports in developing countries rely on traditional means of sharing information regarding cargo, merchant ship documents and invoice-related transactions. Traditional means of sharing documents were based on the internet, emails, and fax. Such processes are susceptible to typing errors and duplicates and are time-consuming (Aydogdu and Aksoy, 2015). Therefore, there is a high risk of data inconsistencies, inefficiencies in information flow and data duplication. According to De Martino and Morvillo (2008), Valentine et al. (2013), Meersman et al. (2014), Carlan et al. (2016) and Moros-Daza et al. (2020), the implementation of PCS can dramatically reduce paperwork, improve data quality, enable data integrity and allow for the efficient flow of information within the supply chain (bills of lading, letter of credit, cargo manifest, dangerous cargo declaration, preannouncements of vessels (ETA/ETD), credit notes et cetera.

The nonexistence of PCS in developing countries deprives port users of real-time information and the prospect of the virtual management of cargo operations. Generally, ports in emerging countries have failed to adopt PCS, placing heavy reliance on traditional means of sharing information regarding cargo. Without them, the ports forgo the opportunity to share information on a single platform. In turn, this phenomenon has exposed ports to the risk of information inaccuracy, duplications, and inefficient flow of information. Consequently, developing countries’ ports have not embraced the opportunity to have an end-to-end view of their logistics chains.

The COVID-19 pandemic and strict country lockdowns worldwide severely affected international trade and South Africa’s economy. The COVID-19 lockdowns and related supply chain challenges resulted in substantial decreases in cargo volumes through South Africa’s ports and significant increases in container freight rates (Grater and Chasomeris, 2022). In 2020, South Africa imposed a strict lockdown, real GDP declined by 6,4 per cent, and annual container trade volumes declined from about 4.59 million TEUs in 2019 to 4.02 million in 2020 (Statistics South Africa, 2022; UNCTAD, 2022). Container terminal productivity was also affected in 2020. The World Bank’s (2021) ranking of 351 competent container handling facilities placed South Africa’s container ports within the bottom five positions, more specifically, the Port of Durban (349), the Port of Ngqura (351), the Port of Port Elizabeth (348) and the Port of Cape Town (347). Such relatively poor rankings alarmed several port stakeholders, including employees of Transnet Port Terminals. As a result, Transnet Port Terminals brought together private and public sector port stakeholders to assess the situation and see what could be done to improve container terminals’ productivity. In South Africa, Transnet National Ports Authority (TNPA) is the single national landlord for the country’s eight commercial ports. TNPA provides the infrastructure and marine services for these ports and has chosen cooperation rather than competition among them. In addition, it dictates the investments to be made and the types of cargo that may be handled at each port (Chasomeris and Gumede, 2022).

The current state of PCS in South Africa’s ports can generally be said to be fragmented, with many handover points resulting in greater time lost on the journey before cargo reaches its destination (Baird, 2002). Port users and stakeholders in South Africa’s ports operate on different information and communication technology platforms (i.e., basically in a silo operation and mentality), which does not allow for the seamless and efficient flow of cargo-related information (De Borger and De Bruyne, 2011). Consequently, there is a great deal of duplication resulting in the wastage of valuable resources, time, money, and human resources (Aydogdu and Aksoy, 2015). Furthermore, observations of the systems used by the five operating divisions of Transnet SOC Ltd., a state-owned logistics company to serve customers, confirm that the divisions are not integrated. The divisions of Transnet conduct business on different information and communications technology (ICT) platforms, although, in several cases, they serve similar or the same customers. This phenomenon results in process duplication, document duplication, and delayed information transfer. It increases business costs and makes the system susceptible to errors due to multiple handover points. Port users in the port system also operate on their different systems with their customers, creating more handover points in the supply chain. Therefore, there is a need for a single window of information and a single information and communication technology platform for managing information and documents between participants to increase port competitiveness (Chamber of Commerce and Industry of Western Australia, 2014).

Subject to this environment, this research aims to collaboratively develop and present a framework for implementing South African port community systems (PCS). It also envisages providing a blueprint for other emerging countries that seek to adopt PCS platforms. Paper-based business transactions are generally a thing of the past. Modern businesses operate on enterprise resource management systems and information and communications technology platforms to facilitate business operations (UNCTAD, 2017). The study uses Soft Systems Methodology to both help understand the status of PCS in ports and unpack the process complexities and contribute to solving port community systems problems in ports. The authors are unaware of previous studies that have used soft system methodology to develop a PCS framework. Therefore, this article contributes to the body of knowledge by applying soft systems thinking to investigate complex matters pertaining to developing a PCS. This article is structured as follows: “Literature review” section presents a literature review on PCS whilst “Research methodology: systems thinking and soft systems methodology” section explains the research methodology employed, specifically soft systems methodology, emphasising the systems-thinking application to the study. “Analyses and findings: applying SSM to establish a framework for South Africa’s port community systems” section focuses on the article’s analysis and findings; finally, “conclusion and recommendations” section presents the conclusions and recommendations.

## Literature review

### Port community systems

There has been considerable change in the 20th Century due to rapid internet development (Lee et al., 2016). Internet-based communication systems have changed how ports do business with their port users (Carlan et al. 2016). Hinterland-based supply chain firms have been using internet-based communication platforms for many years. Major European ports have taken it a step further and have implemented similar systems for sharing information between cargo owners, shipping lines and port authorities (Tijan et al. 2021). This provides one single window for trade, like those found in airport environments. Some developing countries have started moving towards integrated PCS to share important information regarding maritime cargo (Kabui et al. 2019). According to Keceli (2011), some ports operate on terminal-based information systems which are not linked to the broader port communities. Such systems are mainly for terminals to plan and organise terminal-related operations. Terminal-based systems are purely used to secure information regarding the physical location of cargo, planning of ship loading/ unloading and job instructions for the use of terminal equipment (İmre et al., 2011). Due to the complex operations of the ports’ supply chains, stakeholders need to integrate collaborative platforms in the form of PCS (Fedi et al., 2019).

According to Srour et al. (2008), PCS can be defined as holistic, geographically grounded information hubs in global supply chains that primarily serve the interest of a heterogeneous collection of port-related companies. Leonardi & Treem (2012) refer to PCS as neutral and open electronic platforms which enable the intelligent and secure exchange of information between public and private stakeholders to improve efficiencies and competitiveness in the seaport environment. Talley (2014) defines a PCS as a platform for connecting multiple stakeholders and serving as an information hub. Talley also views a PCS as a tool that simplifies the exchange of information regarding commercial and administrative matters to generate added value for stakeholders. Finally, the CCIWA (2014) describes a PCS as a central electronic platform that enables multiple systems operated by different supply chain players to connect and exchange information on one single window platform. PCS are central to ports’ supply chain management in the maritime sector; they have been proven to help improve port efficiencies and attractiveness (Valentine et al., 2013). They encourage and facilitate coordination and cooperation amongst players in the logistics chains. Common platforms are also critical to archiving supply chain efficiencies with the traditional ways of sharing information (Valentine et al., 2013). A PCS connects multiple systems operated by different entities to a single view of information pertaining to the supply chain. PCS platforms are utilised by public agencies and private operators like consigners, consignees, ship owners, ship agents, freight forwarders, cargo owners, port authorities, terminal operators, customs, security agencies, hauliers, and rail operators within maritime and hinterland supply chains in one information-rich system, increasing the competitiveness of the port community (Keceli, 2011). Furthermore, they increase the competitiveness of the port community by coordinating communication procedures within maritime supply chain logistics (De Borger & de Bruyne, 2011). According to Talley (2014), PCS allow supply chain participants to share information efficiently on a single platform by drawing information from different enterprise resource management systems. As electronic platforms, PCS enable the intelligent and secure exchange of information between public and private stakeholders (Chamber of Commerce and Industry of Western Australia (CCIWA), 2014).

PCS platforms enable an end-to-end view of the supply chain (from pit to port) and encourage cooperation between supply chain participants (Celik and Topcu, 2014) and enhances connectedness, contributing to greater competitiveness in the supply chain (Meersman et al., 2014). Trust is fundamental in developing a collaborative platform for supply chain participants (De Martino et al., 2015). According to De Martino and Morvillo (2008) and Moros-Daza et al. (2020), a gap exists in the literature on port community systems and work done in this field is vastly fragmented with few specialists available. This work contributes to closing the already identified gap in literature whilst progressively developing and presenting a framework for port community system implementation. Carlan et al. (2016) suggest that with growing emergent technologies, ICT should be added as an essential strategic pillar to the existing pillars like geolocation, cost and services, political climate, and availability of infrastructure.

### Port community system functionalities and benefits

The main functionalities of the PCS are evident in the electronic communication for efficient management of the entire supply chain (maritime and hinterland) without compromising the privacy of commercial data flowing through the supply chain (Fedi et al., 2019). According to Carlan et al. (2016), many researchers have looked into PCS in recent years. However, very few have attempted to research PCS implementation in ports. The key functions of the PCS revolve around customs clearances, navigational functions, dangerous goods declaration, and port logistics functions like container and truck booking systems. PCS are multi-layered and consist of different functionalities within each layer. The first layer of the PCS consists of functions like booking notifications, customs declarations, and dangerous goods declarations. The second layer incorporates invoicing, berth reservations and safe sea-net notifications. The last layer involves the inland logistics leg, like inland orders and inventory management (Carlan et al., 2016). Carlan et al. (2016) recommended a modular approach to implementing PCS, commencing with its navigational function and dangerous goods module, followed by its terminal booking and customs declaration module and, finally, its in-land order-related module.

There are huge benefits derived from PCS implementation in ports (Srour et al. 2008; Talley, 2014; Carlan et al. 2016). These are mainly economic benefits related to reduced access costs and less paperwork resulting in fewer illegal transactions. The system greatly benefits users in the integrated planning of port logistics, allowing better investment management (Ng et al., 2014). There are also quality-related benefits, such as fewer errors relating to transactions, billing, and quantities. Data accuracy is achievable through suitable port community platforms providing reliable sets of information in real-time (i.e., quick access to critical information, which enhances decision-making). ICT has been regarded as an added pillar in the port system, enhancing the preannouncement of vessel arrivals and facilitating official documents (Meersman et al. 2010). Moreover, according to Meersman et al. (2014), PCSs simplify the control of imports and exports, facilitating better control of administrative processes by customs agencies and port authorities. Indeed, port authorities can benefit from effective, efficient, and seamless traffic flows due to optimised port planning derived from collaborative planning on common platforms. PCS allow for the easy facilitation of dangerous goods/cargo declaration processes (Meersman et al., 2014). Furthermore, PCS enhances efficient resource utilization that would not be possible without a single-window platform for sharing information (Carlan et al. 2016). With PCS in place, the collaboration between stakeholders is much more accessible and user-friendly, making PCS a catalyst for bringing port stakeholders together. PCS allows for better control and monitoring of imports and exports by various stakeholders. As a result, PCS provides a competitive advantage for the ports (Srour et al., 2008).

#### Port community system development

Two fundamental perspectives for examining PCS are exploitative and explorative (Subramani, 2004). PCS contribute to increasing operational efficiencies from an exploitative perspective, whilst the explorative perspective mainly aims to enhance and ensure seamless cargo flow in ports. Although all systems possess attributes of being exploitative and explorative, great care is required during PCS’s implementation when choosing between bilateral, hub or modular systems architecture and whether systems are one-to-many or many-to-many in the case of hub systems architecture (see Table 1).

**Table 1:** Port community system architecture

Generation	Architecture type	Graphics and explanation	
1st Generation	Bilateral (1:1)		The basic form of connection between two trading entities works well for an established partnership (point-to-point)
2nd Generation	Private Hub (1:N)		This architecture makes it possible to connect many entities with few linkages. In addition, the system orchestrates one internal connection point allowing standard access for external partners. This is suitable for larger entities connecting with smaller suppliers
	Central Orchestration Hub (N:1:M)		Like the private hub, the difference is that independent entities operate this system This is best suited for industries where all players are equal
3rd Generation	Modula distributed plug and play (N:M)		This system works on a plug and connects when interactions are required to exchange information or to conduct business. Standardization is the foundation for this architecture

Source: Author adapted from Srour et al. (2008)

Table 1 presents different generations and stages of evolution in the PCS architecture. The older generation of information technology (IT) architecture was merely one-on-one IT platforms. The latest system architecture (Hubs) can accommodate a variety of stakeholders on one platform with fewer connecting points, resulting in greater collaboration between stakeholders (van Baalen et al., 2008). In previous decades cargo-related information arrived in port when the vessel arrived with cargo. Nowadays, this information arrives electronically before the vessel. PCS allows for the integration and automation of information systems linking different stakeholders who collaborate on a single platform (van Baalen et al., 2008). PCS serves a collective goal of intelligently processing and redistributing information pertaining to cargo flow. For this goal to be attained, stakeholders’ commitment, emerging from a common understanding of the salient problems impeding supply chains and a willingness to invest, is fundamental. The scope of implementation should be communicated and agreed upon by all stakeholders involved in the implementation. The following section discusses the critical steps necessary in implementing port community systems in ports.

### Port community systems analysis and design

Port cargo operations and ground-level activities must be transformed into a structure and model, which are moulded into the PCS architecture whilst stakeholders agree on the language to be used. The port community structure, model and architecture should reflect the cargo operation requirements of the flow of information and documents. A suitable PCS should provide users with more than just messaging, and the system should act as a central hub with intelligent decision support functionalities and provide for data integration.

### Implementation and adoption

Implementation of PCS starts when an organization alters its business practices and starts applying new systems and processes to engage with other stakeholders in the supply chain (Srour et al., 2008). The decision by any member of the supply chain to utilize the PCS actively constitutes a system adoption. According to Baron and Mathieu (2013), a PCS should be viewed as an electronic platform that links multiple systems operated by various port community organisations, allowing members to share and exchange information. The gathering of information happens before the merchandise arrives. The electronic platform rapidly improves merchandise coordination at the port level by suppressing paper documents.

Srour et al. (2008) claim that the modular approach is critical when the port community successfully implements PCS. On the other hand, Keceli (2011) recommends that the PCS should be aimed at specific objects common to all port users. Keceli even proposes a three-stage strategy for logically implementing a PCS. The first implementation stage integrates port operators and port authority ICT systems in a single-view window. The second stage of the PCS implementation integrates other information and communications technology systems of other authorities or critical service providers like customs and pilot associations brought into the single platform. The final stage brings into the PCS value-added service providers.

### Maintenance and growth

The maintenance of any system requires ongoing efforts to make the system relevant. According to Srour et al. (2008), it is important for the system to be agile and prepared to adapt to changes to seize emerging opportunities and gain users’ favour as they will increase the utilization of the system. Therefore, the PCS must cater continuously to all port users’ growing and changing needs in the port system.

## Research methodology: systems thinking and soft systems methodology

A qualitative, explorative research approach, namely a soft systems methodology (SSM), was employed to investigate and systematically understand the current status of PCSs in South African seaports, which was considered to be problematic and, after that, to develop a framework for a PCS for South Africa’s ports (Creswell, 2013). The approach was selected to provide the researcher with a systematic approach to collecting and analysing qualitative data (Bryant and Charnaz 2007). According to researcher observation, SSM emerges as the correct systemic way of investigating such problem situations (Checkland and Poulter, 2006). The SSM relies on the concept of ‘system’. A system is a complex whole, the function of which rests on its parts and interactions between them. Examples of these systems can be said to be physical, biological, designs and human activities (Jackson, 2000). Scientists relied on reductionism as a method of studying complex systems. The main focus of the reductionist approach is on the parts as being paramount. The approach seeks to understand the parts and work up from understanding the systems’ parts to the entire system (Jackson, 2000). What was missed by reductionism is that the entire system often exhibits form/shape that is not identifiable by just looking at parts (Capra, 1996). The exhibition of the entire system emerges because of interactions between the parts as parts affect each other through complex networks of relationships (Wiener, 1948).

According to Checkland (1981), the whole system gives meaning to the parts and their interaction; hence the shift away from looking at the system from a reductionist perspective that proved to be limited. The shift to holism was unavoidable as holism views a system as more than just the collection of its parts (Capra, 1996). According to Flood and Jackson (1991), holism focuses on parts and their networks of relationships to understand how they give meaning to the system as a whole because systems are important in studying complex systems. Systems thinking was born out of holism, biology and control engineering combined to become a transdisciplinary approach in studying complex systems (Wiener, 1948; Mingers, 2000). Hard systems thinking was a leap forward in terms of applying systems thinking to real-world problems. However, hard systems thinking was later criticized for its inability to deal with the substantial complexity brought about by plurality, conflicting beliefs, contradictory values, politics, and power at the centre of complex systems (Mingers, 2000). These complexities proved difficult and frustrating for hard systems thinkers as the hard system approach is limited when dealing with wicked problems or messy situations due to its nature which requires the prior identification of concerns (Checkland, 1981). The core of hard systems is optimization in pursuit of a known goal or objective.

## Soft systems methodology

Peter Checkland is the SSM’s founder. He developed it in the 1970s to deal with issues of politics, power, beliefs, values and plurality and published it in the 1980s as an action research method (Checkland, 1981). SSM sets out the principles to engage methods allowing for interventions in ill-structured problem situations involving relationship maintenance instead of goal-seeking (Checkland, 1983). SSM is a systemic methodology that focuses on the whole system rather than on its parts and can accommodate different world views (Checkland, 1981). According to Checkland and Scholes (1990), SSM allows both the analyst and the participants to understand different perspectives of the problem situation, inspiring learning throughout the process of resolving the problem situation. Within the SSM, there are two types of activities: real-world and systems thinking (Checkland, 1987). The SSM models are epistemological devices used to discover the real-world problem (Checkland and Holwell, 1998). Moreover, SSM is interpretive instead of functionalist and based on the understanding that systems are mental constructs produced by observers of the real world. Viewers describe the world differently based on their worldview embodied in the root definitions. Since purpose emanates from the human mind, mental models are important for managing systems, and these models arise from values, understanding, experience and education (Checkland, 1987). Root definitions are the foundation of conceptual models that illustrate the different worldviews (Checkland and Scholes, 1990). Conceptual models are then used to debate the desirable state required to bring about convergence and agreement on the future state. Consequently, SSM facilitates intervention in ill-structured problem situations.

According to Checkland (1981), the analysis phase of SSM should not be to persuade in systems terms as that may lead to the analyst jumping to premature conclusions about the problem situation. Checkland goes on to say that SSM is centred on building a pictorial cartoonish representation of the real problem, referred to as a rich picture and then proceeding to identify a range of complementary systems methods that can be used to improve the problem situation. The purpose of a rich picture is to visually represent activities that humans embark on in pursuit of their purpose, to ignite structured debate pertaining to conflicting values, beliefs, interests, needs and objectives and to bring about collective understanding (Mingers, 1984; Checkland, 1987). Human activity systems can be explored to gather insight into the problem context at this stage. Furthermore, a root definition is formulated considering factors of CATWOE (Customers, Actors, Transformation process, Worldview, Owners, and Environment). When root definitions have been formulated and completed, conceptual models can then be constructed. Finally, conceptual models abstractly describe the physical and social systems or aspects of reality (Checkland and Scholes, 1990). These conceptual models stimulate debate about the real world as they are being compared to the rich picture developed earlier (Checkland, 1983). The comparison process allows discussion about changes needed to improve the problem situation. An agreement amongst those concerned regarding desirable, feasible, culturally, and politically sound change is established and actioned by all parties. Action plans are established to improve the system. The project team is appointed to implement and monitor the implementation of the changes. Once all changes have been implemented, the cycle activates other needs for improvements elsewhere in the system (Checkland, 1987). This takes the shape of a DEMING cycle of continuous improvement, plan, do, check, and act (PDCA) (Moen and Norman, 2010).

## Application of soft systems methodology

As applied to this study, SSM consists of seven stages that are structured sequentially to identify the problem situation, build the model, evaluate the -model, and take action (Bustard et al. 1999). In Stage 1 there is the identification of the problems, Stage 2 expresses the problem situation, Stage 3 presents the root definition, Stage 4 builds the conceptual models, Stage 5 compares the models to the real world, Stage 6 defines the desirable change, and Stage 7 deals with change implementation. As part of implementing stages 1 and 2 of the soft systems methodology process, nonprobability sampling was employed to select participants from different stakeholder groupings (Creswell, 2013). The aim was to select participants who are experts in the ports’ economy and those who are impacted by this problem (Cortina, 2010; Dode et al., 2016). Semi-structured face-to-face interviews were conducted with twelve participants from different port stakeholder groups (TNPA, customs, terminal operators, ship agents, representatives of shipping lines, freight forwarders, stevedores, haulers/ rail operators, experts in port operations and environmentalists). Interviews were recorded, transcribed, categorized and examined to gain a deeper insight into and a richer understanding of the problem situation to assist in constructing a rich picture (Carbin and Strauss, 2008; Checkland, 1983). Finally, the rich picture was presented to participants at the first workshop held on 05/05/2021 to refine the problem situation and begin with stage 3 of soft systems methodology (Root definition).

During workshop 1 discussions of the rich picture, a table of CATWOE elements was formulated to identify Customers, Actors, Transformation process, Worldviews, Owners and Environmental constraints. This study used the CATWOE to formulate a root definition (Checkland, 1981) as follows: a PCS framework that will help organs of the state to develop a common platform for connecting key port users to achieve the seamless, effective and efficient information flow vital for efficient cargo transit in South Africa’s ports. During the first workshop participants discussed key points about stakeholders, document flow, duplicate processes, delays, stages of PCS implementation and funding of PCS implementation. The researcher reviewed the literature to align the project with past research on PCS. A further review of the currently available systems within South Africa’s ports system context was conducted. Continuous examination of the inputs shared by participants was done on an ongoing basis. Data collected from workshop one, the first set of interviews, and random individual contributions were used to develop a second set of semi-structured interview questions. The second round of face-to-face interviews was conducted with twelve participants from port stakeholders, as later indicated. The purpose of the second round of interviews was to construct conceptual models. The second round of interviews was recorded, transcribed, categorized, and analyzed to understand the ideal world as part of stage 4 of the soft systems methodology (Lewis, 2015; Checkland, 1987).

The soft systems methodology facilitated gathering primary data evidence from 24 interviews and two workshops with private and public sector port community stakeholders. The rich-picture and conceptual models were presented at workshop two conducted on 21/06/2021, and this included debates about the ideal world compared to the real world and an attempt was made to agree on the final framework for PCS in South Africa’s ports. The key factors for successful development of PCS framework are stakeholder prioritization, designing of the document flow process and agreeing on the PCS implementation plan. Moreover, participants discussed lead projects and funding models. A detailed discussion and illustrations of the practical application of the soft systems methodology in South Africa’s ports are presented in the Section that follows.

## Analyses and findings: applying SSM to establish a framework for South Africa’s port community systems

*Stages 1 and 2* In Stage 1 of the SSM, a sense of discomfort with the situation considered problematic by members of social groups is identified. The problem situation is narrowed down to a specific problem situation that demands attention. At this stage of SSM, it is crucial to identify the problem situation and express the problem visually in the form of a rich picture. As part of Stage 1, the flow of information and documents between port users becomes a concern due to duplication, inaccuracies, and delays in the system. Mthembu and Chasomeris (2019) also identified this and stated that research into South Africa’s ports’ supply chains is urgently required to investigate inconsistencies in document handling, process duplications, process redundancy, data inaccuracies, and lack of information intelligence. The result of the fieldwork included in the rich picture reflects the participant’s viewpoint regarding the current system in South Africa’s ports. The problem situation presented in the rich picture reflects duplication, redundancy, inaccuracies and lack of information intelligence. The aim is to gain and disseminate a creative understanding of the problem situation (structures, processes at work and relationships).

**Fig. 1:**
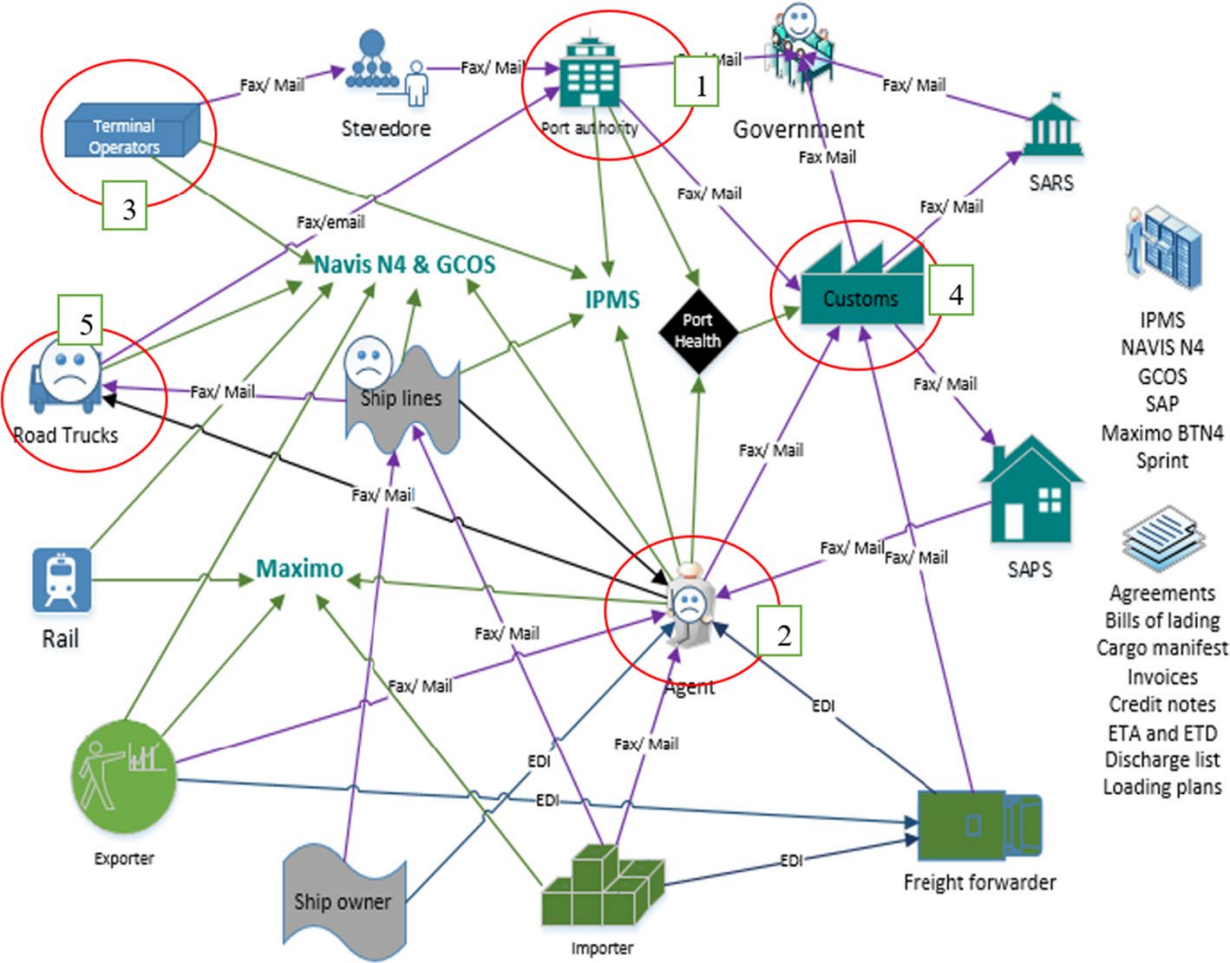
Rich picture of South Africa’s ports community model. Source: Authors compilation from information gathered

Figure 1 illustrates a fragmented port community environment engulfed by individualism and a lack of trust. The picture demonstrates a current complex, disorderly and unintegrated port system prone to delays and errors and the resulting disgruntled customers. There is generally a lack of information integration within South Africa’s port system. Different stakeholders use different information and communication systems to execute similar port operations. The port environment is too manual and fragmented from an information and documentation perspective. Customers are concerned about the current manual system as it is less than ideal. Operations are disintegrated and are observed to be operating in a siloed context with a general lack of trust between port users. The current port environment is susceptible to errors and process duplication. Intermediaries and players utilize different systems to process similar documents. Multiple stakeholders handle documents multiple times in the chain, creating duplications that render the process vulnerable to inaccuracies. The current system of handling information and documents in South Africa’s ports remains complex and cluttered, requiring urgent simplification, decluttering and integration. TNPA, ship agents, terminal operators, customs brokers, and road/rail carriers seem to bear most information and document traffic. This may suggest that they need to be prioritized for the first layer of PCS implementation.

*Stage 3* Stage 3 of SSM works with relevant human activity systems that give insight into the problem; root definitions are constructed from these insights. Table 2 shows CATWOE elements for a port community system in South Africa’s ports. It shows the different stakeholders and their roles in the port community. The most relevant elements of the CATWOE identified as essential for this exercise were key stakeholders immediately affected by the system (port authority, port agent, terminal operators, customs, and road hauliers). Through discussions and interviews, the transformation process system was identified as involving cargo movement through the port system. The environment was viewed as a fragmented system of port management that produces duplication, errors, and delays. Based on the assessment of CATWOE, a root definition was constructed during the second workshop. A root definition is a sentence describing the ideal system citing its purpose, who will be involved in it, who will be affected by it and who can effect change in the system. For example, the CATWOE was used to formulate a root definition (Checkland 1981) as follows: a PCS framework that will help organs of the state to develop a common platform for connecting key port users to achieve the seamless, effective, and efficient information flow vital for efficient cargo transit in South Africa’s ports.

**Table 2:** CATWOE elements for port community system in South Africa’s ports

CATWOE	Port authority (TNPA)	Ship owners	Shipping lines	Ship agent	Terminal operators	Stevedores	Freight forwarder	Customs and excise	Road & rail operators	Police & environment
Customers	Ship agents, ship-owner, cargo-owners, terminal operators, road/ rail operators	Shipping lines, Agents, cargo Owners	Port authorities, Cargo owners, ship agents, freight forwarders	Cargo owners, Terminal operators, Port Authorities	Shipping lines, Ship agents, stevedore, road/rail operators	Ship Agent and Terminals	Cargo owners and Ship agents	Port Authority, Shipping lines, Ship Agents, Freight forwarders	Ship agents, ship lines, cargo owners, freight forwarders	Port Authorities, Customs
Actors	Harbour Master, Port Management	Company Executives	Ship lines Principal and directors	Agents	State (Transnet Port Terminals) and private sector	Private	Private	State	State (Transnet Freight Rail) and private (Road)	State
Transformation process	Administer ports, oversight, enforce rules, improve performance of the ports, ensure the flow of information to all port users	Provision of seaworthy ships to transport merchant shipping goods	Safe transportation of merchant goods around the world	Ensuring the flow of cargo-related documents on time to avoid delays in shipping	Loading and unloading of ships, trucks and rail in port and providing storage for imports and exports	lashing/unlashing, loading/unloading of ships in ports	Movement of cargo from the consignor to consignee safe and on time	Processing of cargo, prevention of illegal goods, monitoring of dangerous goods, arresting crime related to merchant shipping and controlling of stowaways, Revenue collection	Transferring of cargo from ports to hinterland depots and warehouses	Arresting illegal dumping, preventing cargo theft, stopping illegal trade and human trafficking
Worldviews	Provide an effective and efficient port system that caters for inclusiveness	Provide sufficient ships to cater for growing international trade and earn profits	Safely move cargo around the world reliably at a reasonable cost	Berth ships on arrival and ensure swift completion of cargo operation for ships	Security of cargo, efficient loading and unloading of ships in berths, providing sufficient equipment	Fast and safe cargo operation	Cargo to move between consignor and consignee with highest quality and in the best time	Correct declaration of cargo and high level of security in the country, zero smuggling and efficient collection of duties	Efficient flow of cargo and information from ports to hinterland	Safe ports and environment
Owners	State	Private	Private	Private	State and Private	Private	Private	State	State and private	State and NPO
Environmental constraints	State-owned ports that state-owned enterprises manage ensure administration and investment in ports	Ships transporting cargo to global markets, regulated to ensure safety of life at sea and to ensure distribution of merchant trade items with minimum environmental impact	Economic conditions conducive for efficient, effective movement of cargo globally within set cost budgets and quick submission of documents	Quick flow of information about ships and cargo. Availability of equipment in port to service the ship	Availability of ships, cargo and equipment to operate terminals efficiently, ship stowage information on dangerous goods	Availability of ships, competition between stevedoring companies	Access to cargo, congestion in ports	Constraints relating to the flow of cargo information, illegal goads, proper declaration of dangerous goods, prevention of illegal goods and stowaways, as well as a proper collection of duties	On-time availability of information relating to cargo in port, collection and IMDGs	On-time availability of information relating to cargo in port, areas of high risk, illegal goods, improper disposal of harmful waste

Source: Author’s compilation from interviews and port stakeholder committee forums

*Stage 4* The root definition formulated from CATWOE, the rich picture, and face-to-face interviews idealizes a perceived future state within South Africa’s ports. Building from the problem situation expressed using the rich picture and root definition formulated from CATWOE, a conceptual model for South Africa’s port can be constructed. The following assumptions were used in building the conceptual model. 1. All port users understand and agree on the need to implement a port community system. 2. The system’s fragmentation and silo working style produce inefficiencies in the flow of documents and information. 3. Stakeholders had an appetite for funding the development and implementation of the port community system in South African ports. The conceptual model consists of activities with a verb constructed logically to represent activities necessary to achieve the transformation contained in the root definition.

**Fig. 2:**
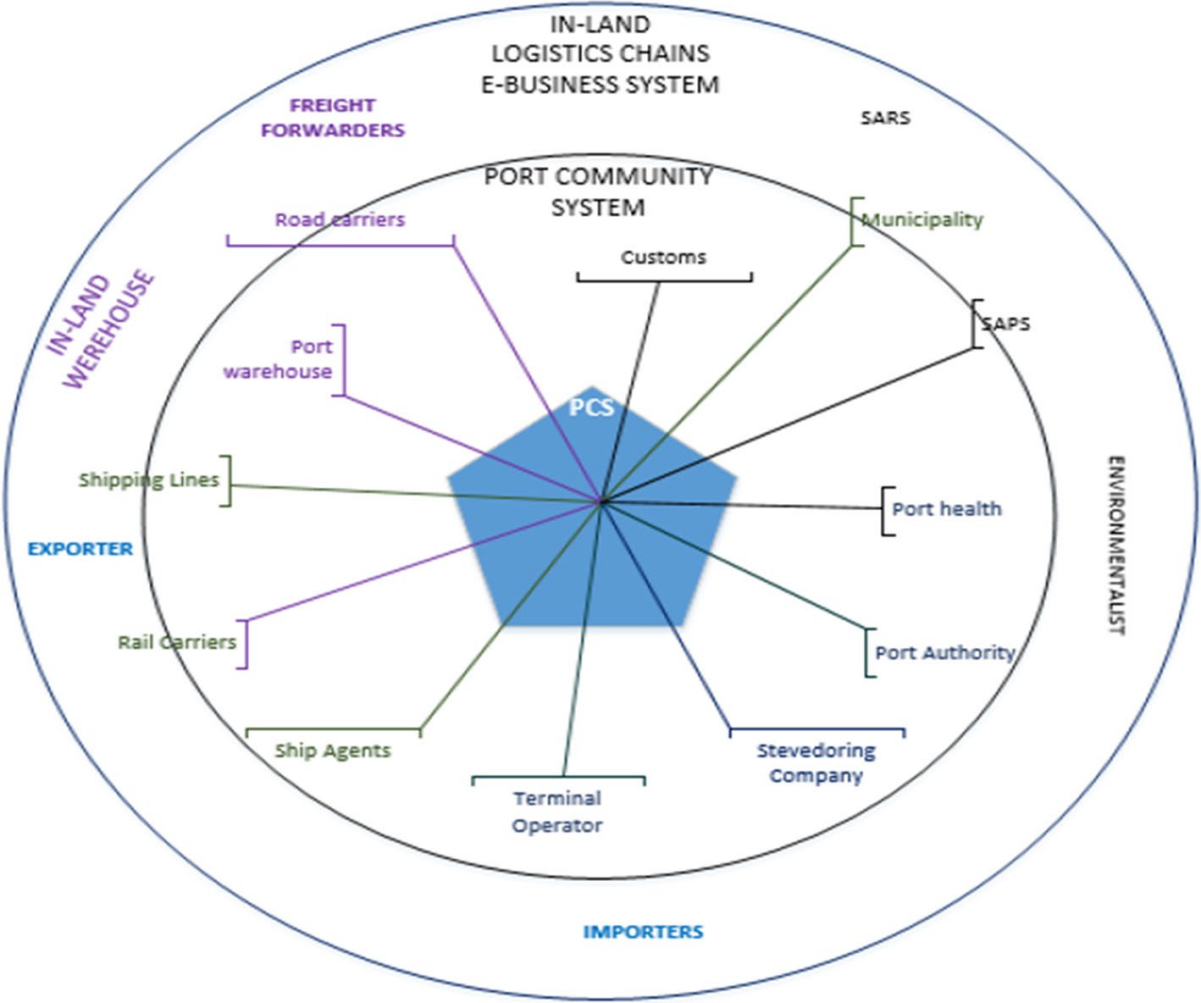
Conceptual model for South Africa ideal world PCS. Source: Author’s compilation using information from interviews and port stakeholder committee forums

Figure 2 illustrates the ideal world suitable and desirable for South Africa’s ports’ context. The conceptual model illustrates a simple, seamless, and integrated port community system for ports with limited risk of duplication and delays. A simplified organizational network seamlessly connected to share important information and documents facilitates cargo flow. The current port community system, compared to the system in the ideal world, magnified the problem situation confronted by the ports in South Africa. Future endeavours must narrow the gap between the current and the ideal world.

*Stage 5* In stage 5 of the SSM, the researcher systematically examined the real-world lack of a PCS in South Africa’s ports and contrasted it with the ideal world PCS as envisaged by the port users during the workshops. Table 3 exhibits the current environment in South Africa’s ports as it emerged from the rich picture and contrasted it with the real-world picture. In Table 3, columns one and two present comparisons of the real and ideal worlds in South Africa’s ports as debated by participants during workshops one and two. Colum three provides the procedure to implement PCS in ports. This is a major contribution of this work. The document sharing is envisaged on a port community system to enable a single view of the supply chain. The system allows participants to have an end-to-end view of the supply chain whilst facilitating real-time decision-making.

**Table 3:** Ideal world versus real world and changes required

Conceptual model activities	Real world	Change action plan
Importer places shipment order from exporter via PCS	Importer places shipment orders via phone, email or fax, manually loaded on Maximo an enterprise resource management system	Appointment of cross-functional and multi-stakeholder teams to drive implementations
Exporter appoints freight forwarder to manage inland logistics—importer, exporter and freight forwarder share information on PCS	The exporter appoints a freight forwarder to manage inland logistics via fax or mail and document shared on EDI	Probe for government support regarding technical infrastructure requirements for PCS
The freight forwarder engages consolidators for container space, shares information on PCS, and engages Ship Agency to charter the ship	Similar information is manually captured on different ICT platforms and sent via emails to different players in the chain	Pursue buy-in from top management of organizations that do business in the ports
The freight forwarder engages with the customs broker regarding information that is shared on PCS	The freight forwarder appoints a consolidator that operates on the different ICT systems. Documents are faxed or emailed	Facilitate discussions regarding PCS with all port stakeholders to consolidate shared benefits
The terminal operator communicates on the PCS stack opening and closing	Freight forwarder appoints inland logistic transporter to manage cargo transfer from inland to ports. Information and documents are manually shared	Categorize port users by their needs and classify them in order of priority
Between freight forwarders and road/ rail carriers, cargo is transferred from the hinterland to ports	The exporter or freight forwarder appoints a ship agent via phone, fax or email to charter the ship on behalf of the exporter	Research and design suitable, reliable and secure PCS to be implemented
Ship agent books the berthing slot from Port Authority on PCS	The ship agent charters the ship, books a slot via IPMS, and arranges cargo operation via NAVIS. Documents are shared via EDI. Contacts stevedoring company manually	Allocate resources and funding for the implementation of PCS
National Ports Authority accepts vessel booking and allocates resources for the vessel’s berthing	Ship agent communicates ETA/ ETD via IPMS with NPA and communicates with the terminal via NAVIS whilst sending documents via EDI	Identify training requirements and train all port users to facilitate the smooth adoption and implementation of the PCS
The ship Agent shares ships’ details, ETA/ ETD, and cargo documents via PCS and the system sends notifications to port health and Systems application and product (SAPS) regarding their visits	Agent arranges stevedoring company and lashing/unlashing gangs via phone, fax or emails. Documents are shared manually	Start implementing the PCS
On vessel arrival, port health and SAPS perform their function; the vessel is docked, and cargo operations commerce and vessel operation progress are shared via PCS to ensure seamless cargo working	Port health and SAPS are informed about ship visits via phone, fax or email	Not applicable
On vessel operation completion, notifications are shared via PCS and the ship sails to the next port of call	Ship agent requests cargo clearance via fax or mail	Not applicable
Not applicable	Cargo booking in and out of the terminal is made on NAVIS, whilst cargo documents are shared via EDI. Between terminal, shipping line and road/ rail carriers	Not applicable
Not applicable	Vessel operation is monitored on NAVIS/ SPARCS container booking system between the terminal, ship agent, or ship line and progress is communicated manually with Port Authority and carriers	Not applicable
Not applicable	Once the cargo operation is completed, ship agent books marine resources via IPMS and confirmation is also via IPMS	Not applicable
Not applicable	Ship sails to the next port of call	Not applicable

Source: Author’s compilation

*Stage 6* The workshop 2 discussion of feasible and desirable changes are produced in column three in Table 3. In the discussion with the participants possible, necessary, systematically desirable, and culturally feasible changes were identified as presented in column three of Table 2. Participants agreed upon the changes. The process employed to identify desirable and culturally feasible changes is consistent with stage 6 of SSM (Checkland, 1987). Compared with the ideal world, the real world led to a debate about required changes that were identified and agreed upon as the organization moves from a fragmented port community system to a more integrated one. Participants discussed a logical approach to implementing the changes, as presented in column three. Table 3 is in line with Checkland’s (1987) three change categories (structure, procedure, and attitudes).

*Stage 7* In stage 7 of the SSM, the researcher examined and consolidated data from the two sets of interviews, two workshops and individual contributions to construct and present a framework for a port community system in South Africa. Table 4 is the second contribution to the study as it presents a framework for building PCS for ports’ layout, stakeholders, functionalities, and documents for PCS. The framework consists of three levels, and the three columns represent stakeholders, system functionality and documents/ information to be shared at various levels of the port community system adoption. The first level of implementation proposes seven stakeholders to be prioritized when designing and implementing PCS (port authority, terminal operator, ship agents, road/rail carriers, and stevedoring companies). The documents shared would include notifications, slot allocations, and declarations. The second level of adoption brings the freight forwarders, shipping lines, port health, warehousing, and law enforcement into the system. Documents to be considered at level two include credit notes, crew declarations, berth reservations, weather detection and port planning. Adopting level three brings on board consignors/consignees, in-land warehousing, municipalities, environmentalists and revenue collection with sharing of documents like orders and custom imports and pre-notifications.

**Table 4:** A framework for implementing PCS in South Africa’s ports

Implementation levels	Stakeholders categories	System functionality	Information and documents
Level one	Port authority Terminal operators Stevedoring Ship agents Customs Road carriers Rail carriers	Booking notifications Slot allocations Customs declarations Dangerous goods	ETA/ETD Cargo manifest IMDG documents Discharge/loading plans
Level two	Shipping lines Freight forwarders Port warehousing Port health Law enforcement	Credit notes Berth reservation Crew declaration Safe sea-net notifications	Bill of lading Agreements Manifest Credit notes
Level three	Consignor/consignees In-landing warehousing Municipality Environmentalist Revenue collection	Inland orders Customs imports Pre-notifications Others	Material requirement plans Production schedules Inventory levels Shipment schedules invoicing

Source: Authors compilation borrowing from Carlan et al. (2016); Srour et al. (2008); Keceli (2011)

## Discussion

The governance, pricing (tariffs) and productivity of South Africa’s ports are highly contested matters by port users and stakeholders (Meyiwa and Chasomeris, 2020; The World Bank 2021). The Ports Regulator of South Africa (2021) publishes an annual port tariff benchmarking study based on a selection of 25 container ports. The exercise used a standardized container vessel to calculate vessel calling costs on 1 April 2020. Of the total cost to move a TEU through a South African port, terminal handling charges contribute 66 per cent, cargo dues contribute 29 per cent, and marine charges contribute 5 per cent. The results show that South Africa’s marine charges are 44 per cent below the benchmarked sample average, but terminal handling charges are 55 per cent above the average and cargo dues are 166 per cent above the average. Total port authority pricing, which includes marine services and cargo dues, is 69 per cent above the sample average. The Ports Regulator of South Africa only regulates TNPA, so the terminal handling charges and productivity of Transnet Port Terminal (TPT) managed container terminals are not under the direct regulation of the Ports Regulator.

The findings show a need to create a PCS in South Africa. Furthermore, the port community stakeholders who were interviewed identified many of the current port supply chain challenges and all stakeholders seem to support the idea of creating a PCS with the associated benefits and responsibilities. Developing and emerging countries’ ports should accelerate the implementation of port community systems converging towards a single window of information for all port users and stakeholders to share information and documents. The planning and execution of operations in South Africa remain fragmented and operate in a silo fashion with a lack of trust among port users. In general, the cost of doing business remains high in ports (Gumede and Chasomeris, 2018; Grater and Chasomeris 2022). By failing to implement PCS, South Africa’s ports have lost the potential benefits and added value attached to the system. These benefits and added values include a high level of services to customers and ships and other benefits relating to cost and operational efficiencies that positively impact ports’ performances (Carlan et al. 2016). Implementing a PCS could facilitate improved information sharing and trade facilitation through which there should be improvements to the supply chain efficiencies that result in enhanced productivity and associated reduction in costs.

Furthermore, the study recommends a practical framework for implementing a PCS in South Africa (see Table 4). In order to develop the framework for implementing a PCS for South Africa (see Table 4), this study reviewed the literature that showed the international precedence, range of benefits and support for the creation of PCS (Srour et al., 2008; Srour et al. 2008; Talley, 2014; Carlan et al. 2016). This study advocates for PCSs to be treated as a critical pillar for ports to be competitive and call for their prioritization in ports. Several valuable examples of well-functioning PCS can be found in Singapore (Portnet and TradeXchange), Hong Kong (One Port and Tradelink), Rotterdam (Port Info Link, Portofrotterdam.com and Webjanas) and Hamburg (Doksy and COAST) among many more (Keceli 2011). Other major European ports have implemented similar systems with a single window for sharing information between cargo owners, shipping lines and port authorities (Tijan et al. 2021).

To achieve greater success during implementation, trust, shared benefits, and stakeholders’ involvement through highlighting shared benefits are essential (De Martino et al., 2015; Vanelslander 2016). According to Srour et al. (2008) and European Commission (2018), trust between port community members is critical if information and communication systems are to be implemented. Figure 1 illustrates a fragmented port community environment engulfed by individualism and a lack of trust. The rich picture in Fig. 1 demonstrates a current complex, disorderly and unintegrated port system that is prone to mistakes and errors resulting in disgruntled customers. A generational evolution in the port community system from one-to-one business to many-to-many business as cited in the modular architecture signifies growth in port community systems (Srour et al., 2008). The soft system methodology adoption of an investigation into the port community system in South Africa yielded the framework for implementing the system. The SSM tools (Rich Picture, CATWOE, Root Definition and Conceptual models) allowed for better facilitation of debates about the most suitable framework for South Africa’s ports (Checkland, 1987). The SSM approach proved suitable for investigating and proposing a framework for South Africa’s PCS.

Stakeholder trust, shared benefits, information security, and continuous system improvement need proper management during and after implementation (Srour et al., 2008; Carlan et al. 2016). As indicated on the change implementation action plan, it is important to appoint cross-functional and multi-stakeholder teams to begin the preparation and development of a port community system implementation plan. The government should drive the implementation of the system in South Africa. The main requirements of implementation are buy-in from top management and support for the system. The source and responsibility for PCS funding in developing countries rests primarily in the government through its key organs (like state-owned enterprises) operating in the port environment (Kabui et al. 2019). In the context of developing countries, it is recommended that the public sector should drive the adoption and implementation of emergent technologies in ports. In developed countries the private sector becomes the driver of adoption and implementation of port community platforms due to high demand and a greater prospect of return on investment. Whilst there is an argument favouring the adoption and implementation, the risk of cyber-attack and protection of information may be a deterrent. Developing PCS policy for developing countries could aid the faster adoption and implementation of the system.

## Conclusion and recommendations

The poor public port governance, high terminal handling charges, high port authority prices and low productivity of South Africa’s container ports as well as other related supply chain issues are matters that are raised by port users and stakeholders. Under such circumstances, the debate around the adoption and implementation of PCS in South Africa is overdue. PCS are central to port supply chains as electronic platforms enable the intelligent and secure exchange of information between public and private stakeholders in ports. They allow supply chain participants to share information efficiently on a single platform and to draw information from different enterprise resource management systems. As a result, PCS can improve productivity, efficiencies, and port competitiveness.

This article reviewed the current port community platforms and used a qualitative soft systems methodology to investigate the current state of PCS and presented a procedure and framework for implementing PCS in South Africa. The SSM tools (Rich picture, CATWOE, Root Definition and Conceptual Models) allowed for better facilitation of debates about the suitable framework for South Africa’s ports (Checkland, 1987). The SSM approach proved suitable for investigating, building, and presenting a framework for implementing PCS. The compelling benefits of implementing PCS are largely argued in the discussion section. The findings showed that South Africa’s ports system stakeholders and port users operate largely in silos. Moreover, there is no single platform for the port community to share standard critical information and documents regarding shipping. Collaboration between supply chain systems’ stakeholders is limited. The existing inefficiencies in the flow of information and documents hamper decision-making in South Africa’s ports. The national government should ensure the availability of infrastructure to support innovations aimed at improving communication platforms between supply chain operators. There is a need to create a PCS in South Africa. The port community stakeholders who were interviewed, support the idea of creating a PCS with the associated benefits and responsibilities. This suggests a high probability for stakeholders’ adoption of the PCS.

The paper presented a framework for developing a PCS and recommended a step-by-step procedure for implementing a PCS in South Africa. PCS have benefits that outweigh the cumulative efforts and costs of research and implementation of a single source of information. Developing and emerging countries’ ports should accelerate the implementation of port community systems converging on a single window of information for all port users and stakeholders to share information and documents. There are shared benefits relating to supply chain efficiencies, traceability, cost optimisation and virtual collaboration that are derived from the adoption of PCS. PCS successful adoption and implementation depends on the willingness to fund both the setup and maintenance of the system. The other critical factors to successful adoption and implementation are the willingness of companies to share information with other parties in the port supply chains. Shared benefits and trust between stakeholders are drivers for adopting and implementing emergent technologies. Developing PCS policy for South Africa and developing countries could aid in faster adoption and implementation of the system. The complexity that emerges from the port being a handover point between land and water operations in ports motivates the desire to exploit information flows for more than mere recordkeeping but for visualizing the port supply chain and gathering intelligence about global trade.

In October 2022, more than a year after our interviews and application of the SSM to develop a framework for implementing a PCS in South Africa, the authors became aware of a recent creation of a Port Community System tool for the Port of Durban that mainly focuses on the container trade. This is a joint initiative coordinated by the South African Association of Freight Forwarders (SAAFF, see: https://saaff.org.za/), Business Unity South Africa and some Transnet divisions. Whilst it is commendable that there is finally an attempt to create a PCS, with funding and data sharing, our preliminary reflections on this initiative reveal some of the merits and demerits connected with attempting to implement this system in practice. Some of the main merits include: a single platform for observing real-time (and historical) data about truck, rail, and ship location (these data are plotted on a map that may help to identify congestion); and movements, like container stack occupancy and slots, truck, rail, and ship productivity measures are recorded. Such information may assist with identifying problems like congestion, measuring productivity, and aiding port users and stakeholders to increase transparency and improve accountability. Some demerits of the system include access to the PCS tool is restricted to members (mainly SAAFF members), and the PCS tool, although a start, is significantly less comprehensive than our proposed PCS framework that is summarized in Table 4. Indeed, our proposed PCS framework (Table 4) and our comparisons between the conceptual model, real-world activities and proposed change action plan (Table 3) may provide some ideas and guidance to SAAFF and other port stakeholders on how to create a more comprehensive PCS. Furthermore, although the information available in the PCS tool is useful for identifying the problems, the resolution of these problems requires active intervention, coordination, dialogue, and investment (both time and money) of the stakeholders to improve the productivity and efficiency of the ports system in South Africa. Future research could focus on quantifying some of the costs and benefits of the proposed PCS as well as developing models for the financing of PCS in emerging and developing economies.

## Data Availability

Authors like to indicate that most data used during the study is available from corresponding author on reasonable request. The data is privately saved on authors password protected laptop. Some data that was used during the study was lost during Transnet system cyber-attack. The IT department could not recover some files as they were corrupted during cyber-attacks. Hard copies of literature reviews are also available on request as they are filed in locked cabin.
